# Targeted Sequencing of Taiwanese Breast Cancer with Risk Stratification by the Concurrent Genes Signature: A Feasibility Study

**DOI:** 10.3390/jpm11070613

**Published:** 2021-06-28

**Authors:** Ching-Shui Huang, Chih-Yi Liu, Tzu-Pin Lu, Chi-Jung Huang, Jen-Hwey Chiu, Ling-Ming Tseng, Chi-Cheng Huang

**Affiliations:** 1 Division of General Surgery, Department of Surgery, Cathay General Hospital, Taipei 106, Taiwan; cshuang@cgh.org.tw; 2 School of Medicine, College of Medicine, Taipei Medical University, Taipei 110, Taiwan; 3 Department of Pathology, Cathay General Hospital Sijhih, New Taipei 221, Taiwan; cy1124@gmail.com; 4 Institute of Epidemiology and Preventive Medicine, National Taiwan University, Taipei 110, Taiwan; tplu@ntu.edu.tw; 5 Department of Medical Research, Cathay General Hospital, Taipei 106, Taiwan; science.man2@gmail.com; 6 National Defense Medical Center, Department of Biochemistry, Taipei 114, Taiwan; 7 Institute of Traditional Medicine, School of Medicine, National Yang Ming Chiao Tung University, Taipei 11121, Taiwan; chiujh@ym.edu.tw; 8 School of Medicine, College of Medicine, National Yang Ming Chiao Tung University, Taipei 11221, Taiwan; lmtseng87@gmail.com; 9 Comprehensive Breast Health Center, Department of Surgery, Taipei Veterans General Hospital, Taipei 1121, Taiwan; 10 School of Public Health, College of Public Health, National Taiwan University, Taipei 100, Taiwan

**Keywords:** concurrent genes, breast cancer, targeted sequencing, actionable mutations, Taiwan, risk stratification

## Abstract

Breast cancer is the most common female malignancy in Taiwan, while conventional clinical and pathological factors fail to provide full explanation for prognostic heterogeneity. The aim of the study was to evaluate the feasibility of targeted sequencing combined with concurrent genes signature to identify somatic mutations with clinical significance. The extended concurrent genes signature was based on the coherent patterns between genomic and transcriptional alterations. Targeted sequencing of 61 Taiwanese breast cancers revealed 1036 variants, including 76 pathogenic and 545 likely pathogenic variants based on the ACMG classification. The most frequently mutated genes were *NOTCH*, *BRCA1*, *AR*, *ERBB2*, *FANCA*, *ATM*, and *BRCA2* and the most common pathogenic deletions were *FGFR1*, *ATM*, and *WT1*, while *BRCA1* (rs1799965), *FGFR2* (missense), and *BRCA1* (rs1799949) were recurrent pathogenic SNPs. In addition, 38 breast cancers were predicted into 12 high-risk and 26 low-risk cases based on the extended concurrent genes signature, while the pathogenic *PIK3CA* variant (rs121913279) was significantly mutated between groups. Two deleterious *SH3GLB2* mutations were further revealed by multivariate Cox’s regression (hazard ratios: 29.4 and 16.1). In addition, we identified several significantly mutated or pathogenic variants associated with differentially expressed signature genes. The feasibility of targeted sequencing in combination with concurrent genes risk stratification was ascertained. Future study to validate clinical applicability and evaluate potential actionability for Taiwanese breast cancers should be initiated.

## 1. Introduction

Breast cancer is the most common female malignancy in Taiwan. Treatment outcomes have improved enormously in the past decade, mainly with the wide spread of screening mammography and efficient systemic therapies [1,2,3]. Currently adjuvant therapies for breast cancer are determined based on staging and pathological factors such as estrogen receptor (ER) and human epidermal growth factor receptor II (HER2) status. These factors not only guide treatment selection but also predict therapeutic responsiveness.

These clinical and pathological factors, however, do not provide full explanation of prognostic heterogeneity within each breast cancer subgroup [4]. For example, one-fourth of HER2 overexpressed breast tumors eventually develop resistance to trastuzumab, a humanized monoclonal antibody to HER2 protein [5]. Sanger sequencing, gene expression (GE), and single-nucleotide polymorphism (SNP) microarrays have surveyed cancer genomes, including sequence variants, DNA copy number variation (CNV), loss of heterozygosity (LOH), and whole transcriptome, leading to the discovery of several molecular taxonomies, many of which have shown prognostic ability [6,7]. The Cancer Genome Atlas Network (TCGA) demonstrated that GE-based intrinsic subtypes displayed alterations across tumor DNA, DNA methylation, messenger RNA (mRNA), microRNA, and protein expression hierarchy [8]. Breast cancer is heterogeneous in terms of molecular aberrations. Oncogenesis may originate from single-nucleotide variations (SNVs) and chromosomal structure abnormalities such as CNVs and may present phenotypically as GE and protein expression profiles [9]. However, the relationships across DNA sequences, mRNA transcription, and protein translation are not always linear and are intervened through complex regulatory mechanisms. It is speculated that cancer results from the progressive accumulation of genetic aberrations. Amplified regions contain dominant oncogenes, whereas deleted regions harbor tumor suppressor genes.

It is not a coincidence that our published breast cancer concurrent genes signature was based on genes with coherent patterns between chromosomal and transcriptional variations [10]. Concurrent genes were identified through genome-wide characterization of Taiwanese breast cancers by integrating comparative genomic hybridization (CGH) and GE microarrays. Genes with concurrent gains and losses from the same subject may be better candidates to compose prognostic biomarkers. A breast cancer risk predictive model was built with distinct survival patterns observed between high- and low-risk group [11]. The risk score was significantly higher for breast cancer patients with recurrence, metastasis, or mortality than those remained disease-free (0.241 versus 0, *p* < 0.001).

The massive parallel sequencing, or next-generation sequencing (NGS), is advocated for parallel sequencing of whole genome, exome, or transcriptome with enhanced accuracy and efficiency without a priori sequence knowledge [12]. On the other hand, targeted sequencing is especially suitable for solid tumors to identify somatic mutations associated with therapeutic sensitivity or resistance. Most targeted agents, whether in development or post-marketing, are portrayed to act against proteins and/or pathways commonly perturbed by tumor genetic changes. Thus, there remains an urgent need to identify actionable mutations for wide clinical application of personalized and precision medicine [13].

The aim of the study was to perform NGS in combination with breast cancer concurrent genes signature. Somatic mutations with clinical significance were identified. Accurate risk assessment is essential for breast cancer effective treatment. This study evaluated the feasibility of integrating targeted sequencing with gene expression-based risk stratification. Clinically actionable mutations and predicted risk groups were evaluated for enrolled Taiwanese breast cancers.

## 2. Materials and Methods

### 2.1. Overall Aims

**Figure 1:**
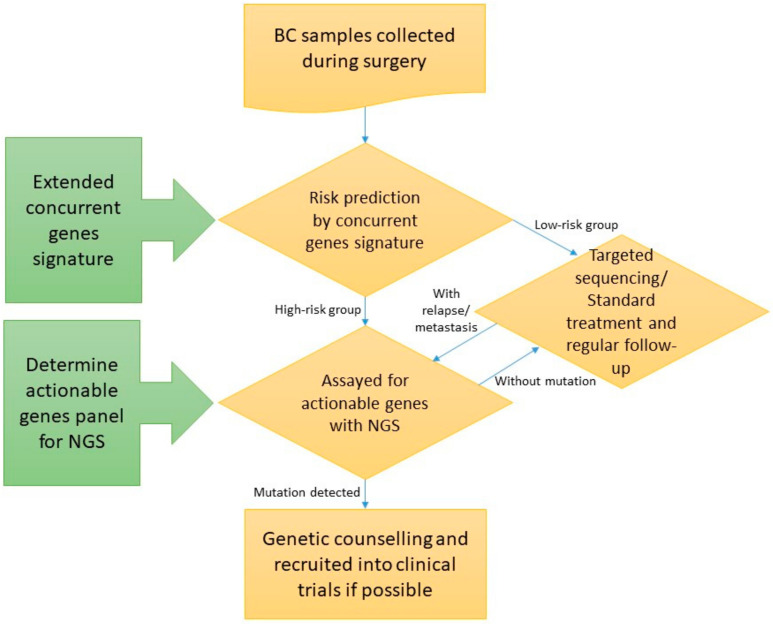
Schematic flow chart of the study (BC: Breast cancer; NGS: Next-generation sequencing). Enrolled subjects underwent targeted sequencing, and those who were also experimented with extended concurrent genes signature were predicted into high- and low-risk group.

We evaluated the feasibility of integrating targeted sequencing and the extended concurrent genes signature [10]. Enrolled subjects underwent targeted sequencing of actionable mutations, and those who were also experimented with GE assays were predicted into high- and low-risk group by the extended concurrent genes signature. The variants of the whole cohort, as well as the predicted high- and low-risk groups, were reported and compared. A schematic flowchart of the study is delineated in Figure 1. The study protocol was reviewed and approved by IRB of Cathay General Hospital with written informed consent obtained from all participants. Significantly mutated genes between the high- and low-risk breast cancers were revealed, and the interaction between tumor genomics and transcriptome was deciphered through identifying variants-associated differentially expressed signature genes.

#### 2.1.1. Breast Cancer Sample Recruitment

Breast cancer samples were collected during surgery, snapped frozen in liquid nitrogen, and stored under −80 °C between 2010 and 2014. Frozen samples were dissected into slices of 1–2 mm thickness, and more than 70% of cancerous content was required. Clinical information and follow-up status were ascertained from Cancer Registry through subjects’ ID. Survival data were censored on 30 November 2019. Regarding pathological features, ER positivity was defined as at least 10% of nuclei staining positive with immunohistochemistry (IHC) assay, and patients with low ER positivity (1–9% of nuclei with positive staining) were not recruited. For HER2 status, the ASCO/CAP guidelines were adopted. IHC 3+ and IHC 2+ with fluorescence in-situ hybridization (FISH) amplification were considered HER2 overexpression. All pathological diagnoses were ascertained by a qualified pathologist (CYL).

#### 2.1.2. Nucleic Acid Extraction for GE Microarray and NanoString nCounter

Nucleic acid extracted from fresh samples was used for microarray experiments. Total RNA was extracted from frozen specimens by TRIzol reagent (Invitrogen, Carlsbad, CA, USA). Purification of RNA was performed with the RNeasy Mini Kit (Qiagen, Germantown, MD, USA) according to manufacturer’s instructions. RNA integration was checked by gel electrophoresis, while 2 bands of 18s and 28s represented satisfactory RNA quality.

Additional samples underwent NanoString nCounter (NanoString Technologies, Inc., Seattle, WA, USA) as described elsewhere [14]. In brief, total RNA was isolated from formalin-fixed, paraffin-embedded (FFPE) sections. RNA was quantified by the Qubit RNA HS Assay Kit (Thermo Fisher Scientific, Waltham, MA, USA), with quantity/quality determined by NanoDrop spectrophotometers (Thermo Fisher Scientific) using a wavelength spectrum of 220—320 nm, evaluating the 260/280 ratio, and by separation on an Agilent BioAnalyzer 2100 (Agilent Technologies, Inc., Santa Clara, CA, USA).

#### 2.1.3. Nucleic Acid Extraction for Targeted Sequencing

Archived pathological slides were retrieved from Department of Pathology. Paraffin blocks with cancer cells composing less than 70% of the section area were excluded, and paraffin was removed by xylene extraction then by ethanol washes. Tumor DNA was extracted from 10-μm sections using a High Pure FFPET DNA Isolation Kit (Roche Applied Science, Indianapolis, IN, USA) with contaminated RNA removed by RNase. DNA purity was verified by the Bioanalyzer, and DNA quality control was indicated by OD260/280 > 1.8. The amount of extracted DNA was quantified by the NanoDrop ND-1000 Spectrophotometer (Wilmington, DE, USA).

#### 2.1.4. Extended Concurrent Genes Signature

Our previous study had identified concurrent genes signature, highlighting the implication between CNV and GE for Han Chinese breast cancers [10]. An updated version of the extended concurrent genes signature has been described elsewhere, with more samples and public domain GE datasets incorporated to enhance generalizability and prognostication [14,15]. A brief description is given here. First, 31 CGH and 83 GE microarrays were performed, with 29 breast cancers assayed from both platforms. Potential targets were revealed by Genomic Identification of Significant Targets in Cancer (GISTIC) from CGH arrays [16]. Concurrent genes and genes with significant GISTIC scores were used to derive signatures. Signatures obtained consensus from leading-edge analysis across all studies, and the supervised partial least square (PLS) regression predictive model of disease-free survival was constructed [17].

#### 2.1.5. Actionable Genes for Targeted Sequencing

Candidate actionable genes for targeted sequencing were determined in a priori manner with the requirement of having been reported as breast cancer driver mutations, coinciding with known or potential therapeutic agents, or being considered actionable through bioinformatics analysis.

#### 2.1.6. Library Preparation and NGS Experiments

The Agilent HaloPlex Target Enrichment System (Agilent Technologies, Inc.) for Illumina Hiseq (Illumina, Inc., San Diego, CA, USA) paired-end sequencing was used for library preparation. Tumor DNA was digested in 8 different restriction reactions (225 ng DNA/reaction), with each containing 2 restriction enzymes. All restriction reaction results were validated using the Agilent 2200 TapeStation (Agilent Technologies Inc.) with High Sensitivity D1K ScreenTape (Agilent Technologies Inc.). The collection of DNA restriction fragments was hybridized to the HaloPlex probe capture library (54 °C, 3 h). The circularized target DNA-HaloPlex probe hybrids were captured on streptavidin beads (HaloPlex Magnetic Beads, Agilent Technologies Inc.) and added DNA ligase to close nicks in the hybrids. Captured DNA was eluted with NaOH, and the cleared supernatant was transferred 20 μL from each tube to a PCR Master Mix tube held on ice. Target libraries were amplified through 22 cycles of PCR, and the PCR product was purified using Agencourt AMPure XP beads (Beckman Coulter, Brea, CA, USA). Finally, all samples were sequenced on Illumina NextSeq500 (Illumina, Inc.) using 150PE protocol.

#### 2.1.7. Variant Annotation and Statistical Analysis

The sequences generated from NGS went through a filtering process to obtain qualified reads. Trimmomatics was implemented to trim or remove the reads according to the quality score [18]. The qualified reads data then went through a genomic alignment against hg19 using BWA to obtain basic sequence information [19].

For further interpretation of NGS results, variants calling and annotation were performed by SureCall (Agilent Technologies Inc.), Partek Flow (Partek Inc, St. Louis, MO, USA), and Ion Reporter (Thermo Fisher Scientific) for visualization. All statistical analyses were performed using SAS statistical software (SAS Inc., Cary, NC, USA). Continuous variables were summarized as the number of observations, mean, standard deviation, and 95% confidence interval (CI). Categorical variables were presented as counts and percentages. Unless otherwise specified, all statistical assessments were performed at the significance level of 0.05.

#### 2.1.8. Data Availability

Raw data of individual targeted sequencing in fastq format will be deposited in NCBI Sequence Read Archive (SRA, submitted BioProject: PRJNA731998) and will be publicly available once the manuscript is accepted.

## 3. Results

### 3.1. Actionable Genes for Targeted Sequencing

The targeted sequencing panel was based and modified from the ClearSeq Cancer (Agilent Technologies Inc.), which was a target enrichment panel designed specifically for known genetic anomalies and cancer hotspots (Table S1). The original application targeted a set of genes found to be associated with a broad range of cancer types, functionally annotated with dbSNP, as well as therapeutic options with the COSMIC database as the primary reference [20]. ClearSeq Cancer was also compatible with HaloPlex and the HaloPlex HS Target Enrichment System (Agilent Technologies Inc.). In the current version, there were 56 targets (genes) comprising 990 regions with a total size of 173.999 kbp, resulting in a coverage rate of 99.75%.

### 3.2. Breast Cancers Assayed for Targeted Sequencing

**Figure 2:**
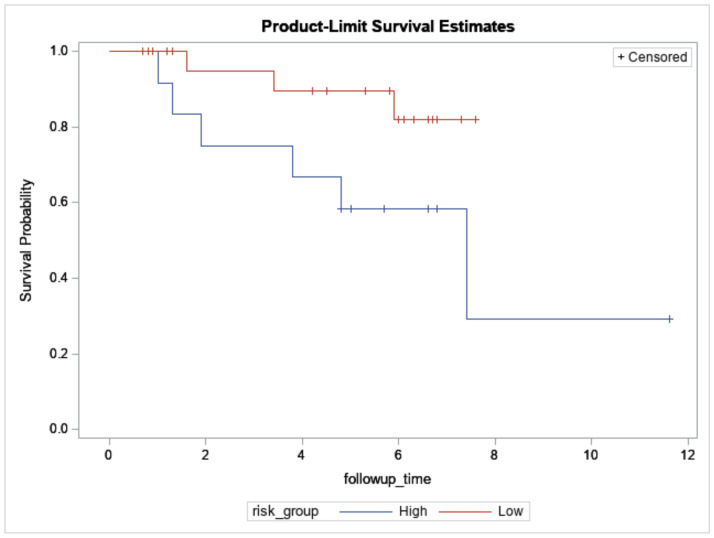
Overall survival of 12 high- and 26 low-risk Taiwanese breast cancers predicted by the concurrent genes signature who also underwent targeted sequencing. X-axis: Follow-up time in years. Y-axis: survival probability.

In total, 61 breast cancers underwent NGS (Table 1). Among them, 38 breast cancers were also assayed with Affymetrix microarrays (Thermo Fisher Scientific, *n* = 33) or NanoString nCounter (NanoString Technologies Inc., *n* = 5) and were predicted into 12 high-risk and 26 low-risk cases based on the extended concurrent genes signature [14,15]. The median follow-up was 3.4 years (range: 0.1 to 11.6 years), and 11 relapses and 10 all-cause mortalities (including 6 breast cancer-specific mortalities) were observed during this period. Figure 2 showed overall survival between the predicted high- and low-risk groups (log-rank test: *p* = 0.06).

Post-alignment QA/QC, including alignments breakdowns, average alignments per read, and average base quality per position and read, were checked and satisfactory (data not shown). Targeted sequencing of 61 Taiwanese breast cancers revealed 1036 variants, including 76 pathogenic and 545 likely pathogenic variants based on the ACMG (American College of Medical Genetics and Genomics) classification [21]. The most frequently impacted genes were *NOTCH, BRCA1, AR, ERBB2, FANCA, ATM*, and *BRCA2*, harboring 57, 36, 30, 27, 27, 26, and 26 variants, respectively. The most common pathogenic deletions were *FGFR1, ATM*, and *WT1* (47, 47, and 37 patients, respectively), while *BRCA1* (rs1799965, nonsense mutation, p.C197C), *FGFR2* (missense mutation), and *BRCA1* (rs1799949, nonsense mutation, p.S694S) were the most common pathogenic SNPs (44, 35, and 11 patients, respectively, Table S2). Under stringent pathogenicity defined by dbSNP database, whole cohort analysis showed that the most common mutations were *ERBB2* rs28933370 (p.N857S), *PIK3CA* rs121913279 (p.H1047L/R/P), and *BRCA2* deletion (p.I605fs*9), impacting 46, 8, and 6 patients, respectively [22] (Table 2).

**Figure 3:**
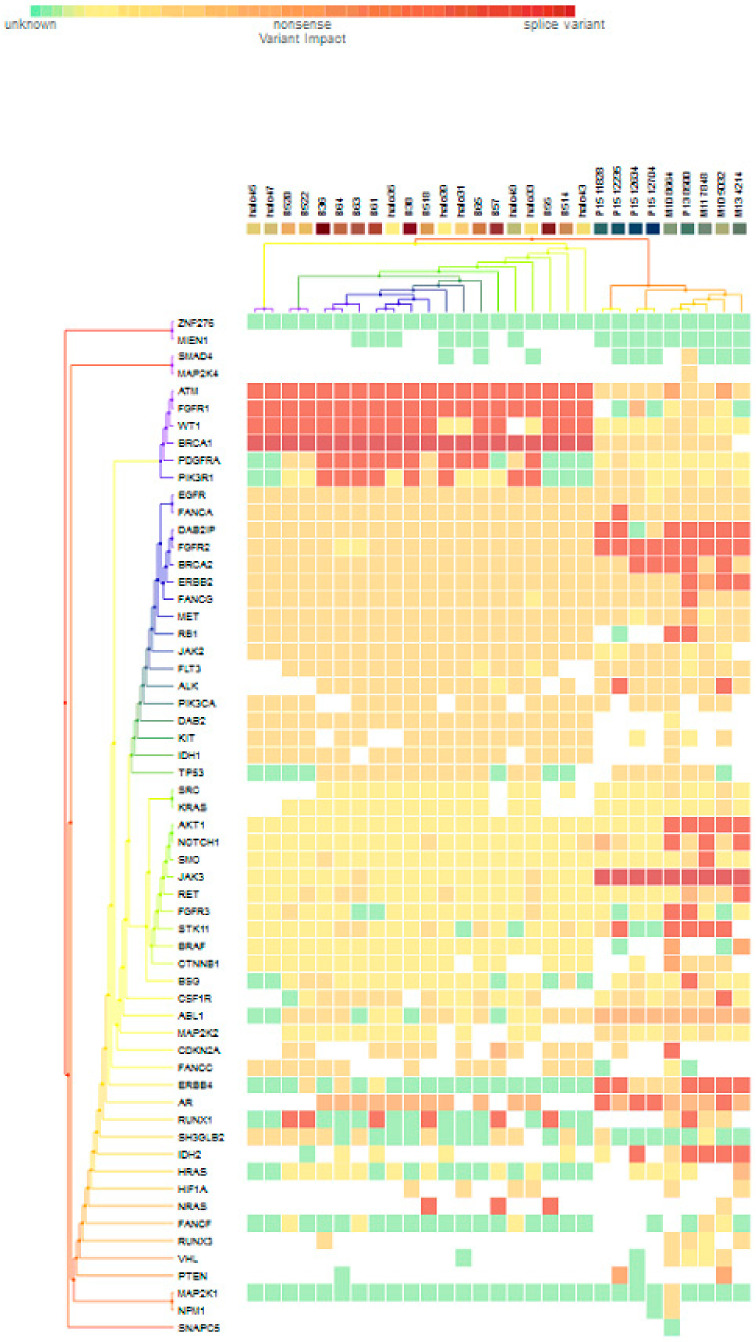
Heat map for 31 HR+/HER2− subtype breast cancers. The variants impacted for 31 HR+/HER2− breast cancers were INDELs. Frameshift: *ATM, FGFR1*(20), *WT1*(16), *PDGFRA*(10), *PIK3R1*(8), *FANCA*(1), *DAB2IP*(7), *FGFR2*(9), *BRCA2*(5), *ERBB2*(3), *FANCG*(1), *RB1, ALK*(2), *AKT1*(5), *NOTCH1*(3), *SMO, RET*(1), *FGFR3*(2), *STK11*(5), *BSG*, *CSF1R*, *CDKN2A*(1), *ERBB4*(6), *AR*(4), *RUNX1*(7), *IDH2*(5), *NRAS*(3); non-frameshift: *FGFR1*(1), *NOTCH1*(1), *RET*(1), *ABL1*(9), *CDKN2A*(1), *AR*(16), *HRAS*(1); SNPs splice: *BRCA1*(20), *JAK3*(9); missense: *SMAD4*, *MAP2K4*(1), *ATM*(7), *FGFR1, WT1*(2), *BRCA*(8), *PDGFRA*(5), *PIK3R1*(6), *EGFR*(28), *FANCA*(28), *DAB2IP*(21), *FGFR2*(18), *ERBB2*(25), *FANCG*(27), *MET*(26), *RB1*(23), *JAK2*(24), *FLT3*(22), *ALK*(21), *PIK3CA*(22), *DAB2*(20), *KIT, IDH1*(19), *TP53*(18), *SRC*(2), *KRAS*(1), *NOTCH1*(5), *SMO*, *JAK3*(2), *RET*(7), *FGFR3*(2), *STK11(6), CTNNB1(3), BSG(5), CSF1R(13), ABL1(10), MAP2K2(4) CDKN2A(12), FANCA(13), ERBB4*(4), *RUNX1*(3), *SH3GLB2*(12), *IHD2*(1), *HF1A*(6), *NRAS*(1), *FANCF*(1), *RUNX3*(4), *VHL*(2), *MAP2K1*(1), *NPM1*(1); and nonsense: *ATM*(2), *ERBB2*(1), *MET*(1), *BRAF*(2), *CTNNB1*(1), *PTEN*(2).

**Figure 4:**
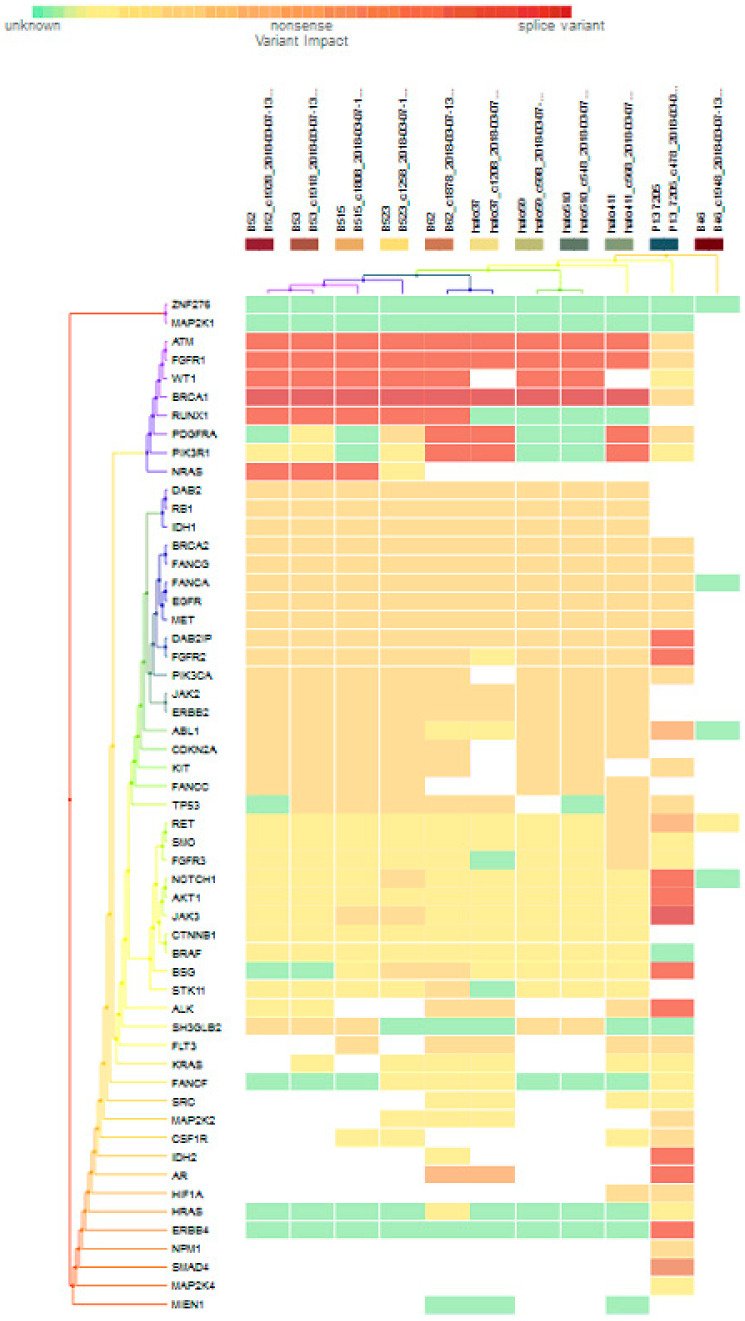
Heat map for 11 HR+/HER2+ subtype breast cancer. The variants impacted for 11 HR+/HER2+ breast cancers were INDELs. Frameshift: *ATM, FGFR1*(10), *WT1*(7), *RUNX1*(5), *PDGFRA, PIK3R1, NRAS(3), DAB2IP, FGFR2*, *NOTCH1, AKT1, BSG, ALK, IDH2, AR, ERBB4*(1); non-frameshift: *ABL1*(1), *RET*(1), *AR*(2); SNPs splice: *BRCA1* (9), *JAK3*(1); stop loss: *SMAD4*(1); missense: *ATM*, *FGFR1*, *BRCA1*, *PDGFRA*(2), *DAB2, RB1, DH1*(9), *BRCA2, FANCG, FANCA, EGFR, MET*(10), *DAB2IP*(9), *FGFR2*(8), *PIK3CA, JAK2, ERBB2* (9), *ABL1*(7), *CDK2NA, KIT*(8), *FANCC, TP53*(7), *RET, SMO, FGFR3, NOTCH1*(1), *JAK3, BSG*(2), *STK11*(1), *ALK*(3), *SH3GLB2, FLT3*(5), *MAP2K2, CSF1R*(1), *HIF1A*(2), *NPM1*(1).

**Figure 5:**
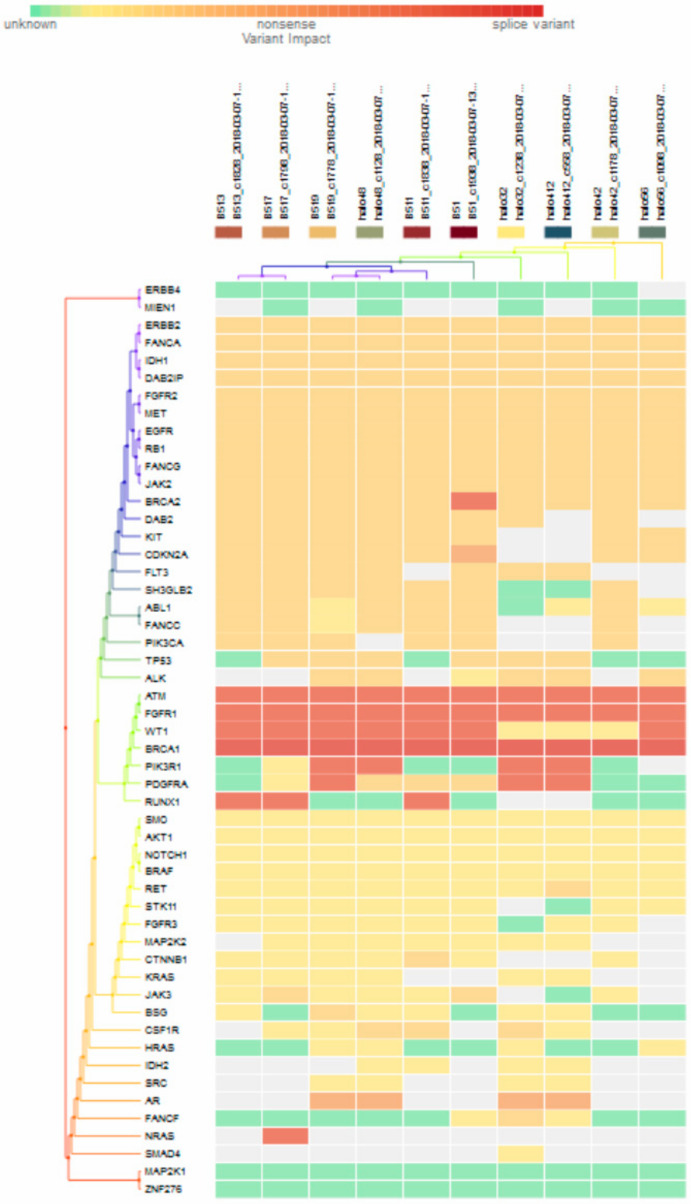
Heat map for 10 HR−/HER2+ subtype breast cancer. The variants impacted in 10 HR-/HER2+ breast cancers were INDELs. Frameshift: *BRCA2* (1), *ATM, FGFR1*(10), *WT1* (7), *PIK3R1* (4), *PDGFRA* (3), *RUNX1*(3), *NRAS*(1); non-frameshift: *CDKN2A* (1), *AR*(4); SNPs splice: *BRCA1* (10); missense: *ERBB2, FANCA, IDH1, DAB21P, FGFR2, MET, EGFR, RB1, FANCG, JAG2* (10), *BRCA2* (9), *DAB2, KIT* (8), *CDKN2A, FLT3, SH3GLB2* (7), *ABL1, FNACC, PIK3CA, TP53* (6), *ALK* (5), *PDGFRA* (3), *RET*(1), *CTNNB1*(1), *JAK3*(2), *BSG*(1), *CSF1R*(3), *FANCF*(1).

**Figure 6:**
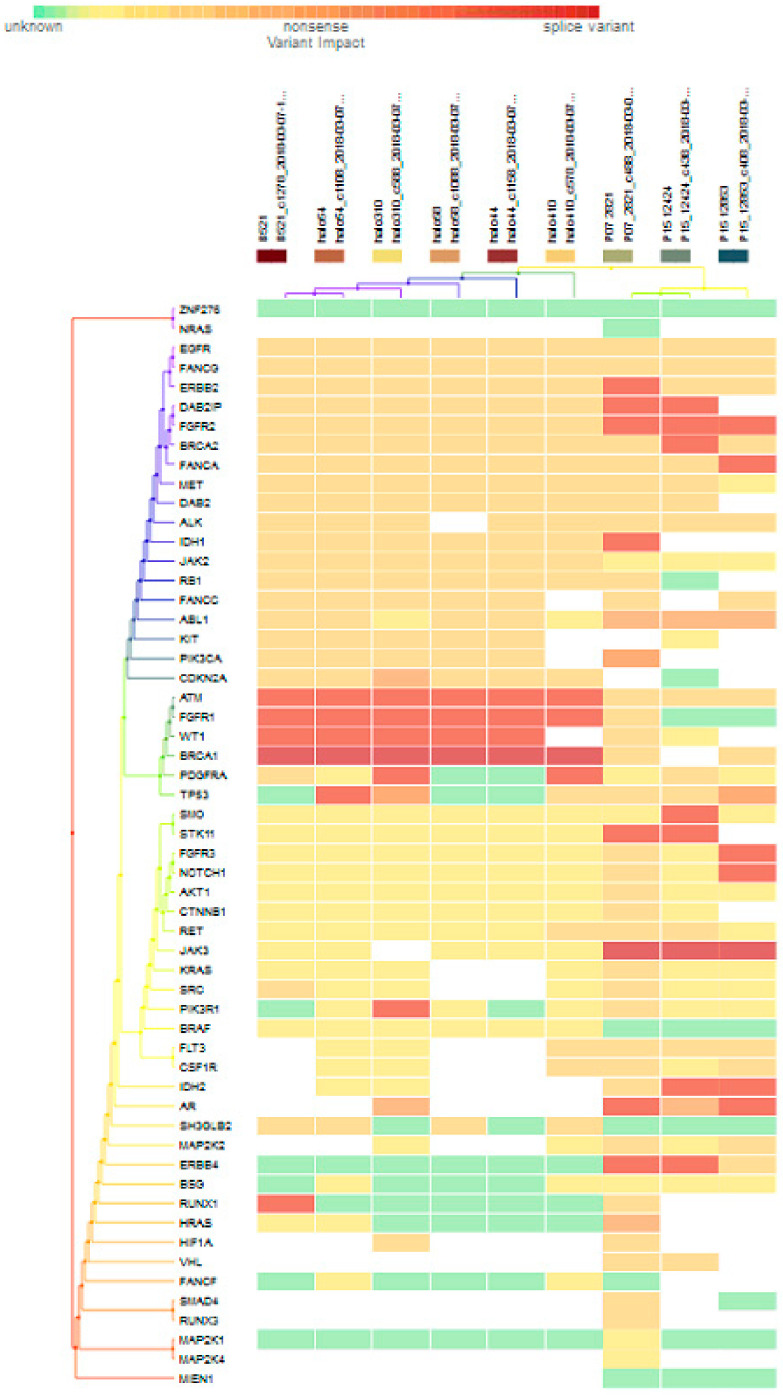
Heat map for 9 HR-/HER2- subtype breast cancer. The variants impacted in 9 HR-/HER2- (TNBC) were INDELs: *ERBB2* (frameshift: 1), *DAB2IP* (frameshift: 2), *FGFR2* (frameshift: 3), *BRCA2* (frameshift: 1), *FANCA* (frameshift: 1), *IDH1* (frameshift: 1), *ATM1* (frameshift: 6), *FGFR1* (frameshift: 6), *WT1* (frameshift: 5), *PDGFRA* (frameshift: 2), *TP53* (frameshift: 1), *SMO* (frameshift: 1), *STK11* (frameshift: 2), *FGFR1* (frameshift: 1), *NOTCH1* (frameshift: 1), *PIK3R1* (frameshift: 1), *IDH2* (frameshift: 2), *AR* (frameshift: 2, non-frameshift: 2), *ERBB4* (missense: 2), *RUNX1* (frameshift: 1), *HRAS* (no-frameshift: 1), and SNVs: *BRCA1* (splicesite_5: 6), *TP53* (nonsense: 2, missense: 3), *FGFR3* (missense: 1), *NOTCH1* (missense: 1), *AKT1* (missense: 1), *CTNNB1* (missense: 1), *RET* (missense: 3), *JAK3* (splicesite_3: 3), *KRAS* (missense: 1), *SRC* (missense: 2), *PIK3R1* (missense: 1), *FLT3* (missense: 4), *CSF1R* (missense: 3), *IDH2* (missense: 1), *SH3GLB2* (missense:4), *MAP2K2* (missense:2), *ERBB4* (missense:1), *RUNX1* (missense: 1), *HIF1A* (missense: 2), *VHL* (missense: 2), *SMAD4* (missense: 1), and *RUNX3* (missense: 1).

For subgroup analyses, variant impacts were color-coded using the associated score values with the heat map of each IHC subtype reported in Figure 3, Figure 4, Figure 5 and Figure 6. Default scores are unknown: 0, synonymous: 1, missense: 2, non-frameshift block substitution: 3, non-frameshift insertion/deletion: 4, nonsense: 5, stop-loss: 6, frameshift block substitution/insertion/deletion: 7 and splice variant: 8. For 31 hormone receptor (HR)+/HER2− breast cancers, there were two clusters: One with the co-occurrence of *ATM, FGFR1*, and *WT1* frameshift mutations, and the other with *JAK3* splice site and *FGFR2* frameshift mutations (Figure 3). The mutational profile of HR+/HER2+ displayed a major cluster of *ATM, FGFR1*, and *WT1* frameshift mutations (*n* = 10 for *ATM1, FGFR1* and *n* = 7 for *WT1*, Figure 4). The heatmaps of the 10 HR−/HER2+ breast cancers were homogeneous (Figure 5), while for 9 HR-/HER2− cases, a major cluster with *ATM, FGFR1*, and *WT1* frameshift mutations (n = 6 for *ATM1, FGFR1* and *n* = 5 for *WT1*, Figure 6) and a minor one with *JAK3* splice site mutations were prominent (*n* = 3, Figure 6). Supplementary File S1 contained a MAF file with all indicated variants.

### 3.3. Significantly Mutated Genes between the High- and Low-Risk Breast Cancers

Since transcriptome-based risk stratification was of major interest in the current study, we compared mutation profiles between breast cancers predicted into the high- and low-risk groups. There were 21 variants, collapsed into 14 genes, including 8 pathogenic/likely pathogenic variants (7 missense, 5 silent SNPs and 1 deletion, collectively 5 genes). *PIK3CA* rs121913279, with amino acid changes p.H1047L, p.H1047R, and p.H1047P, was reported as being pathogenic from dbSNP (Table 3). Among them, *PIK3CA*, *PDGFRA*, *CSF1R*, *EGFR*, *SH3GLB2*, *ATM*, *ERBB2*, *BRCA1*, *BRCA2*, and *MAP2K2* were more likely to mutate in high-risk patients. Multivariate Cox’s regression was performed for these differentially mutated genes with forward selection, and two SH3GLB2 variants with amino acid change p.V223M were deleterious, with hazard ratios of 29.4 (95% CI: 5–173.9, *p* < 0.001) and 16.1 (95% CI: 2.7–96.7, *p* < 0.001) reported after controlling for clinical ER, HER2, and grade.

### 3.4. Variants-Associated Differentially Expressed Genes

**Figure 7:**
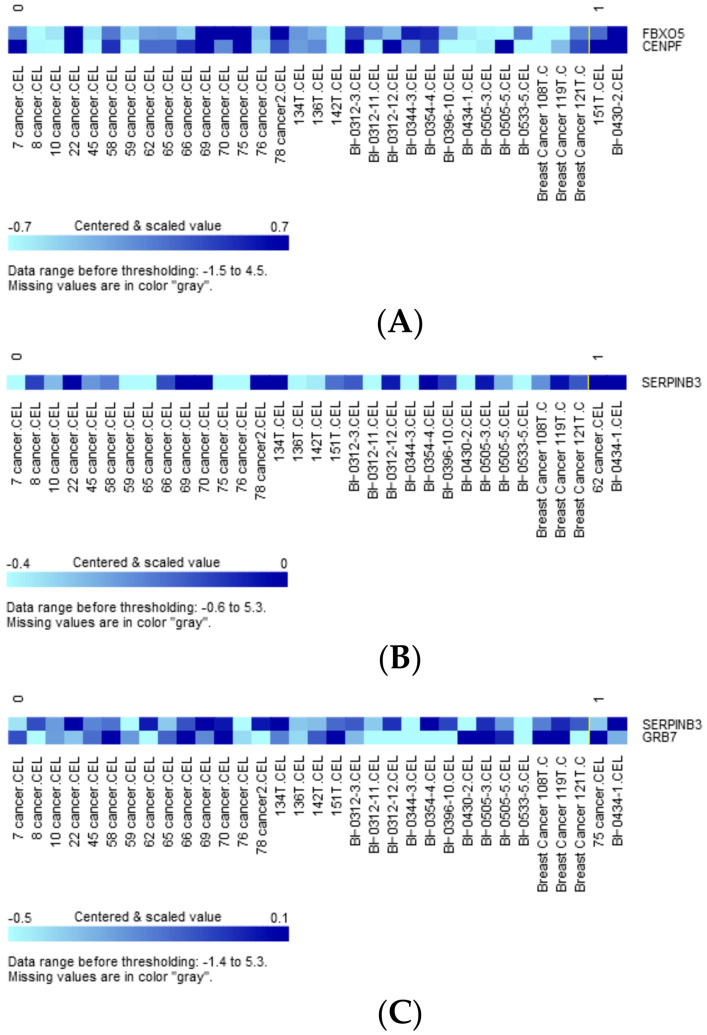
(**A**) Clustered heatmap of differentially expressed genes *FBXO5* and *CENPF*, with samples grouped by class. Class 0: *SH3GLB2* wild-type (31 samples), class 1: *SH3GLB2* mutant-type (2 samples). (**B**) Clustered heatmap of differentially expressed gene *SERPINB3*, with samples grouped by class. Class 0: *SH3GLB2* wild-type (31 samples), class 1: *SH3GLB2* p.V223M (2 samples). (**C**) Clustered heatmap of differentially expressed genes *SERPINB3* and *GRB7*, with samples grouped by class. Class 0: *ERBB2* wild-type (31 samples), class 1: *ERBB2* p.P107L (2 samples).

We also evaluated genes whose expression was differentially impacted by tumor mutations. Variants significantly mutated between the predicted high- and low-risk group (Table 3) as well as those categorized as oncogenic, predicted, or likely oncogenic by OncoKB were used as grouping variables [23]. Two-sample t-tests were conducted for each of 48 constitutional genes of the extended concurrent genes signature under equal or unequal variances assumption based on the equality of variances test with a reduced α level of 0.01 corrected for multiple comparisons. Table S3 shows the exhaustive results of 48 gene-level transcriptions tabulating 56 preselected variants. Expression of *FBXO5* and *CENPF* was significantly upregulated with *SH3GLB2* mutation (Figure 7A, parametric *p*-values: 0.0003153 and 0.007465, respectively, both FDR < 0.2), while *SERPINB3* expression was significantly upregulated by *SH3GLB2* p.V223M mutation (Figure 7B, parametric *p*-values: 8.9e-06, FDR: 0.000427). Two patients harboring *ERBB2* p.P107L reported greater upregulated expression of *SERPINB3* and *GRB7* than wild-type (Figure 7C, parametric *p*-values: 6.72e-05 and 0.0087323, both FDR < 0.2). Other variant-transcription combinations with insignificant trends included: *BSG* mutation upregulated *UBE2V2* expression (*p*-value: 0.0045538, FDR:0.219), *BSG* p.D181D mutation downregulated *LACTB2* expression (*p*-value: 0.0087388, FDR > 0.2), *ERBB2* p.L120 upregulated *SERPINB3* expression (*p*-value: 0.0054249, FDR: 0.26), and *FANCA* p.*A430* downregulated *KIF14* and *CENPF* (*p*-values: 0.002285 and 0.0095128, FDR: 0.11 and 0.205).

## 4. Discussion

In current study, we evaluated the feasibility of targeted sequencing combined with gene expression signature for Taiwanese breast cancers. Traditionally, multigene expression signatures are used as a prognostic tool to identify a subset of low-risk breast cancer patients who might be spared cytotoxic chemotherapy, especially for luminal (HR+/HER2−) breast cancers. NGS, especially the tumor-only targeted sequencing, was performed to reveal actionable mutations corresponding to novel therapeutics. The combination of gene expression-based prognostication and NGS-based predictive biomarkers was appraised for Taiwanese breast cancers.

The merit of the extended concurrent genes signature was the discovery of candidate biomarkers not readily identified by conventional GE-only data, for which phenotype-correlation or gene variability was the criteria of gene filtering [10]. On the other hand, high-throughput parallel massive sequencing could identify large numbers of variants depending on both the size of the sequenced regions and the variant caller algorithm utilized. For example, Meric-Bernstam et al. reported the experience with 2000 consecutive patients with advanced cancers who underwent NGS, including the frequency of actionable alterations across tumor types and subsequent enrollment into clinical trials [24]. Breast cancer is among one of the most common cancer types diagnosed and assayed, constituting one of the major components of completed comprehensive genomic sequencing.

Initially, an updated list of actionable genes for Taiwanese breast cancers was pursued. Extensive literature reviews have shown some potential candidates. Arnedos et al. reported targeted genomic alterations for metastatic breast cancers and highlighted that identification of DNA damage repair (DDR) defects and mechanisms of immune suppression were potential uses of genomics for personalize medicine [25]. The development of precision medicine for the treatment of breast cancer has several major challenges, including the low frequency of targetable molecular alterations, feasibility of high-throughput technologies, and availability of approved or investigated targeted therapy. Relling, M.V. and Evans, W.E. also pointed out that somatically acquired variants might direct the choice of targeted anticancer drugs for individual patients [26].

In contrast to whole-genome/exome sequencing, targeted sequencing was adopted in current study, allowing the identification of somatic alterations for breast cancer pathogenesis. Although whole-genome sequencing was feasible, we preferred targeted sequencing of specific actionable genes for current task. Targeted sequencing was more affordable, yielded much higher coverage of genomic regions of interest and reduced sequencing cost and time [27]. The merit of targeted sequencing comes from the fact that these panels sequence only desired regions and eliminate most of the genome from analysis. Consequently, these panels encompass hotspots for cancer-driver or relevant mutations, and the identification of disease-targeted alterations could aid in therapeutic decision-making in breast cancer therapy [28]. The ClearSeq Cancer platform is especially suited for clinical samples such as preserved FFPE archives. Highly fragmented DNA usually results in insufficient sequencing target coverage during FFPE preparation, while HaloPlex covers each base with several amplicons and produces smaller fragments function as a backup for longer fragments that might fail [29]. This allows for adequate sequencing target coverage, even in highly degraded FFPE samples.

There were several recurrent aberrations reported from the 61 Taiwanese breast cancers. The *ERBB2* rs28933370 missense mutation was reported as being pathogenic/likely pathogenic from ovarian cancer with somatic allele origin, while neither ascertain criteria nor alternative allele frequency from 1000 Genomes or the Taiwan Biobank were provided [30,31]. Consequently, the clinical significance of this variant remains unknown for Taiwanese patients. *FGFR1* amplification (8p12) is one of the most common focal amplifications in breast cancer (around 10%), especially for the ER-positive phenotype. Overexpression of *FGFR1* is induced by cyclin D1 via the pRb/E2F pathway, while cyclin D1 is overexpressed in human malignancies and correlates with poor prognosis [32]. As an oncogene, *FGFR1* deletion is less understood, while *FGFR2* (10q26) missense mutation may be indicative of anti-FGFR2 inhibitor, as amplification or overexpression of *FGFR2* was observed in 4% of triple negative breast cancers [33]. Some activating somatic point mutations have been reported for both *FGFR1* and *FGFR2*, which couple with an aberrant signaling in a ligand-independent manner such that oncogenic activity exerts by amplification or overexpression. One *FGFR3* rs121913112 pathogenic mutation with germline allele origin was also identified in current study. Although most *FGFR* SNPs remain variants of unknown significance (VUS) under current knowledge, future studies to elucidate their roles in disease susceptibility, prognosis (germline origin), and expressed quantitative trait loci (eQTL) regulating cis/trans gene expression (somatic origin) are warranted during biomarker discovery.

The *BRCA2* p.I605fs*9 deletion, impacting six breast cancers in current study, was predictive of treatment response of PARP (poly ADP ribose polymerase) inhibitors based on the OlympiAD and EMBRCA trials. This deletion could have also been also predictive of synthetic lethality if these advanced/metastatic breast cancers were HER2 negative and germline mutations were ascertained from reflex testing as well [34,35]. On the other hand, the two nonsense *BRCA1* rs1799949 and rs1799965 mutations, although categorized as pathogenic by SureCall, were synonymous from dbSNP database, which partially explains the high prevalence among the Taiwanese population as evidenced by the much higher minor allele frequency (MAF) of 0.38 for rs1799949 (Taiwan Biobank). The *PIK3CA* rs121913279 (p.H1047R and p.H1047L) was among the hotspot mutations indicative of the use of alpelisib, a PI3Kα-specific inhibitor while Figure 3 showed that 22 out of 31 HR+/HER2- breast cancers harbored *PIK3CA* missense mutations [36]. Quite a few *TP53* pathogenic mutations (annotated with germline origin) were also reported in the current study. However, it is worth noting that *TP53* variants rarely represent germline Li-Fraumeni syndrome. Thus, routine reflex germline testing was not necessary for most patients with tumor-only sequencing [37]. *ATM* deletion was also recognized as being pathogenic, as the DDR signaling pathway was orchestrated by both *ATM* and *ATR* kinases, which play the central regulatory role of this network, while the clinical significance of somatic *ATM* mutation requires further evaluation [38]. *PIK3R1* was also pathogenic, which is the regulatory subunit p85α of the PI3K pathway, and somatic loss of *PIK3R1* might be sensitive to MAPK inhibitor [39]. It has also been reported that *PIK3CA* and *PIK3R1* mutation is mutually exclusive, leading to oncogenesis and hyperactivity of PI3K pathway [40].

Figure 3 to Figure 6 show clustering heat maps of 61 Taiwanese breast cancers based on the scores 0 to 7, with higher weights designated for worse pathogenicity. Although heat maps of each IHC subtype were constructed separately, the limited sample size prevented pairwise comparisons. Co-occurrence of *ATM*, *FGFR1*, and *WT1* frameshift mutations were observed across all subtypes, which might reflect targeted panel design. At least two clusters constituted both HR+/HER2− and HR−/HER2− subtypes, indicating more heterogeneous molecular aberrations.

There were eight pathogenic/likely pathogenic variants from five genes differentially mutated between predicted high- and low-risk breast cancers with the extended concurrent genes signature. Of note, *PIK3CA* p.H1047L and p.H1047R were pathogenic and predictive of alpelisib-targeted therapy [36]. Two *SH3GLB2* variants (one with p.V223M amino acid change), which encoded endophilin-B2 and interacted with *SH3GLB1* and *SH3KBP1*, were hazardous for overall survival [41]. Less is known about *SH3GLB2*, but endophilin B2 has been reported to facilitate endosome maturation [42].

As both targeted sequencing and GE data were available in some subjects of current study, it was quite intuitive to investigate the interaction between cancer genomics and transcriptome. As shown in Figure 7A–C, both *SH3GLB2* variants upregulated *FBXO5*, *CENPF,* and *SERPINB3*, with the latter also being upregulated by *ERBB2* p.P107L mutation, which also enhanced the transcription of *GRB7*. These trans regulations from significantly mutated genes (two *SH3GLB2* variants) and OncoKB-defined oncogenic *ERBB2* mutation further highlight the complex network between genomic and transcriptional aberrations, and the necessity of discovering biomarkers hierarchically. *SERPINB3* overexpression is associated with high-grade, HR-negative breast cancers and poor survival [43]. *GRB7* locates in the long arm of chromosome 17 next to *ERBB2*, while co-amplification and co-expression of these two genes have been described [44]. Increased expression of *FBXO5* has been shown to cause chromosomal instability and cancer initiation [45]. It is noteworthy that a lack of evidence remains to directly claim that genetic mutation of A causes the transcriptional alteration of gene B, and it is highly possible that the transcriptional alteration of gene B is a secondary (indirect) effect of genetic mutation in A. Further functional assays are required to elucidate the complex regulatory mechanisms.

Some limitations of the study should be considered. First, for some recurrent mutations, such as those from *CTNNB1*, *CSF1R*, *JAK2*, *HRAS*, and *RUNX1*, an exhaustive literature search showed that the clinical significance in breast carcinoma has rarely been addressed. Second, tumor-only sequencing was performed, and it was difficult to differentiate somatic mutations from those with germline origin, which might hinder the clinical applicability, particularly for PARP inhibitors, as germline mutation of *BRCA1* or *BRCA2* is the prerequisite for synthetic lethality. Third, not all cases of targeted sequencing underwent GE assays due to limited fresh frozen or FFPE samples, and the estimated mutation frequency might be biased. Although our previous study had ascertained measurement invariance between microarray and NanoString nCounter, uniform and unbiased GE assays are required in further studies [14]. To translate sequencing results into clinical actionability, the most difficult aspects to overcome are accurate functional annotation, reproducibility, and the immediate implementation of identified variants.

## 5. Conclusions

Precise risk assessment is fundamental following the diagnosis of breast cancer. The current study provides real-world evidence regarding the feasibility of targeted sequencing combined with concurrent genes signature risk stratification. Targeted sequencing of actionable genes is believed to provide clinical applicability, and future studies with more prospectively enrolled samples are believed to provide substantial benefit for breast cancer patients in terms of precision medicine. The purposed integrated approach could identify potential therapeutic targets, which, in turn, would enhance breast cancer risk prediction to identify subjects for whom increased risk of relapses or metastases will balance discomfort and complications induced by adjuvant chemotherapy and/or targeted therapy. On the other hand, those predicted with lower risk might be spared from potential harms of adjuvant therapy. The current study provides real-world evidence regarding the feasibility of such an approach, and future prospective studies are needed.

## Figures and Tables

**Table 1:** Clinical features of 61 Taiwanese breast cancers with targeted sequencing.

Sample ID	Follow-Up Time (Year)	Relapse Status *	Vital Status *	Age	Stage	Predicted Risk Group	HR	HER2	Grade
#36	0.9	0	1	68	0		1	0	3
#38	0.7	0	1	55	1A		1	0	1
#46	4.8	1	1	47	3A	High	1	1	3
#51	1.3	0	0	58	4	High	0	1	3
#52	6.8	0	1	55	3C	Low	1	1	3
#53	6.7	0	1	34	1	Low	1	1	2
#55	5.0	0	1	47	0	High	1	0	2
#57	6.7	0	1	57	1	Low	1	0	2
#61	0.8	0	1	48	3A	Low	1	0	3
#62	3.9	1	0	56	3A		1	1	3
#63	0.8	0	1	55	2B		1	0	3
#64	0.9	0	1	61	2A	Low	1	0	2
#65	0.7	1	1	67	3C	Low	1	0	2
#511	6.1	0	1	38	2A	Low	0	1	2
#513	6.0	0	1	55	1	Low	0	1	2
#514	5.8	0	1	44	0	Low	1	0	3
#515	5.7	0	1	47	2B	High	1	1	3
#517	4.2	0	1	52	0	Low	0	1	3
#518	4.5	0	1	48	1	Low	1	0	2
#519	1.3	0	1	42	2B	Low	0	1	3
#520	7.4	0	0	74	1	High	1	0	3
#521	1.9	0	0	44	2A	High	0	0	3
#522	4.8	1	0	39	4	High	1	0	2
#523	5.3	0	1	53	2B	Low	1	1	2
#31	1.0	0	1	45	2B		1	0	3
#32	1.0	1	0	46	4	High	0	1	3
#33	11.6	1	1	50	3B	High	1	0	3
#35	0.9	0	1	70	2A		1	0	2
#37	0.8	0	1	51	1A		1	1	1
#39	0.8	0	1	44	1A		1	0	2
#41	5.8	0	1	50	1	Low	1	0	3
#42	1.6	1	0	50	4	Low	0	1	3
#43	7.6	0	1	33	2B	Low	1	0	1
#44	7.3	0	1	54	3A	Low	0	0	3
#45	5.9	1	0	69	2B	Low	0	0	2
#47	3.4	1	0	42	2A	Low	1	0	3
#48	3.8	0	0	57	2B	High	0	1	3
#49	1.2	1	1	57	999	Low	1	0	2
#54	6.8	0	1	43	2A	High	0	0	3
#56	6.6	0	1	46	2B	High	0	1	3
#58	6.8	0	1	45	2A	Low	0	0	3
#59	6.6	0	1	46	1	Low	1	1	2
#310	0.8	0	1	59	1A		0	0	3
#410	0.8	0	1	59	1A	Low	0	0	3
#411	0.8	0	1	50	3C	Low	1	1	3
#412	0.9	0	1	64	1A		0	1	3
#510	6.3	0	1	69	2A	Low	1	1	2
#512	6.1	1	1	61	2A	Low	1	0	2
#13	1.0	0	1	47	1A		1	0	2
#16	0.8	0	1	46	0		1	0	2
#19	0.3	0	1	56	1A		1	0	1
#18	0.9	0	1	45	2A		1	0	2
#14	5.3	0	1	39	2A		0	0	3
#12	0.1	0	1	62	3C		1	1	2
#110	6.0	0	1	63	0		1	0	1
#11	1.0	0	1	37	1A		1	0	1
#21	0.8	0	1	58	3A		1	0	1
#22	0.9	0	1	46	1A		0	0	3
#23	0.8	0	1	60	2A		1	0	1
#24	0.9	0	1	88	1A		1	0	2
#25	0.9	0	1	50	2B		0	0	3

* Relapse status (0: Disease-free, 1: Relapse), vital status (0: Dead, 1: Alive).

**Table 2:** Pathogenic variants identified by dbSNP database.

Gene	refSNP ID	Type	Function Class	Cosmic Amino Acid Syntax	Impacted Patients
*ERBB2*	rs28933370	SNP	MISSENSE	p.N857S	46
*PIK3CA*	rs121913279	SNP	MISSENSE	p.H1047L,p.H1047R,p.H1047P	8
*BRCA2*		Deletion		p.I605fs*9	6
*TP53*	rs11540652	SNP	MISSENSE	p.R248Q,p.R248L,p.R248P,p.R155Q,p.R155P,p.R155L	3
*CTNNB1*		SNP	NONSENSE		1
*FGFR3*	rs121913112	SNP	MISSENSE		1
*CSF1R*		SNP	MISSENSE		1
*JAK2*	rs77375493	SNP	MISSENSE	p.V617F,p.V617I,p.V617_C618 > FR	1
*HRAS*	rs104894228	SNP	MISSENSE	p.G13R,p.G13S,p.G13C	1
*TP53*		SNP	NONSENSE	p.R306*	1
*TP53*	rs28934578	SNP	MISSENSE	p.R175H,p.R175L,p.R43H,p.R82H,p.R82L,p.R175P,p.R43L	1
*RUNX1*		SNP	MISSENSE	p.H85N	1

**Table 3:** Variants significantly mutated between the predicted high- and low-risk group of Taiwanese breast cancer.

Gene	Mutation Type	refSNP ID	ACMG Category	Function Class	*p*-Value (χ^2^-Test)
*PIK3CA*	SNP		Category II	MISSENSE	0.03
*PIK3CA*	SNP	rs121913279	Category II	MISSENSE	0.02
*PDGFRA*	SNP	rs35597368	Category II	MISSENSE	0.01
*CSF1R*	SNP		Category III		0.02
*EGFR*	SNP		Category III	SILENT	0.02
*MET*	SNP	rs41736	Category III	SILENT	0.05
*FGFR1*	SNP		Category III		0.01
*SH3GLB2*	SNP		Category III		0.02
*SH3GLB2*	SNP		Category II	MISSENSE	0.02
*ATM*	Deletion		Category I		0.04
*BRCA2*	SNP	rs56403624	Category II	MISSENSE	0.02
*BRCA2*	SNP	rs169547	Category II	MISSENSE	0.04
*FANCA*	SNP		Category III	SILENT	0.01
*FANCA*	SNP		Category III		0.04
*ERBB2*	SNP		Category III	SILENT	0.02
*ERBB2*	SNP		Category II	MISSENSE	0.02
*BRCA1*	SNP	rs55946644	Category III		0.01
*BSG*	SNP		Category III		0.02
*BSG*	SNP		Category III	SILENT	0.04
*BSG*	SNP		Category III	SILENT	0.01
*MAP2K2*	SNP	rs10250	Category III	SILENT	0.04

## Data Availability

Raw data of individual targeted sequencing in fastq format are deposited in NCBI Sequence Read Archive (SRA, submitted BioProject: PRJNA731998).

## Supplementary Materials

The following are available online at https://www.mdpi.com/article/10.3390/jpm11070613/s1, Table S1: Gene panels of targeted enrichment sequencing. Table S2: Pathogenic mutations affecting more than 10 Taiwanese breast cancers. File S1.zip: 61_targeted_seq.maf for all called variants and Sample_Tumor_Sample_Barcode_convertion.xlsx for sample ID and sample barcode conversion. Table S3: Two sample t-tests of 48 gene-level transcriptions grouped by 56 preselected variants with equal/unequal variances assumption based on equal variances testing and reduced α level for multiple comparisons.
