# Supplementary material for: Targeted Sequencing of Taiwanese Breast Cancer with Risk Stratification by the Concurrent Genes Signature: A Feasibility Study
Source: J Pers Med. 2021 Jun 28;11(7):613. doi: 10.3390/jpm11070613 (PMC8306786; doi:10.3390/jpm11070613)
Supplement: Supplementary file 1 [file jpm-11-00613-s001.zip › Supplementary materials/Table S1 and S2.pdf]

Table S1. Gene panels of targeted enrichment sequencing.

Gene symbol	Regions	Coverage	High coverage	Low coverage
ABL1	14	99.75%	14	0
AKT1	14	100%	14	0
ALK	31	100%	31	0
AR	11	100%	11	0
ATM	62	99.78%	61	1
BRAF	21	100%	21	0
BRCA1	24	100%	24	0
BRCA2	28	99.63%	28	0
BSG	10	100%	10	0
CDKN2A	5	100%	5	0
CSF1R	22	100%	22	0
CTNNB1	14	100%	14	0
DAB2	13	100%	13	0
DAB2IP	18	100%	18	0
EGFR	32	99.92%	32	0
ERBB2	29	99.78%	29	0
ERBB4	29	100%	29	0
FANCA	46	97.64%	44	2
FANCC	15	100%	15	0
FANCF	1	100%	1	0
FANCG	14	100%	14	0
FGFR1	21	100%	21	0
FGFR2	23	100%	23	0
FGFR3	18	100%	18	0
FLT3	25	100%	25	0
HIF1A	16	100%	16	0
HRAS	5	100%	5	0
IDH1	8	100%	8	0
IDH2	11	100%	11	0
JAK2	23	100%	23	0
JAK3	23	100%	23	0
KIT	22	100%	22	3
KRAS	6	100%	6	0
MAP2K1	12	100%	12	0
MAP2K2	11	100%	11	0

MAP2K4	14	100%	14	0
MET	21	100%	21	0
NOTCH1	34	100%	34	0
NPM1	12	100%	12	0
NRAS	4	100%	4	0
PDGFRA	24	100%	24	0
PIK3CA	20	98.61%	18	2
PIK3R1	19	100%	19	0
PTEN	9	100%	9	0
RB1	27	100%	27	0
RET	20	98.86%	19	1
RUNX1	12	97.03%	11	1
RUNX3	7	100%	7	0
SH3GLB2	15	100%	15	0
SMAD4	13	100%	13	0
SMO	13	100%	13	0
SRC	12	100%	12	0
STK11	9	100%	9	0
TP53	14	97.88%	13	1
VHL	3	98.58%	3	0
WT1	11	100%	11	0

Table S2. Pathogenic mutations affecting more than 10 Taiwanese breast cancers.

Gene	Mutation type	Effect	Affected subjects	Pathway
FGFR1	Deletion	FRAME_SHIFT NM_001174063	47	PI3K, FGF signaling
ATM	Deletion	FRAME_SHIFT NM_000051	47	DNA damage response
WT1	Deletion	FRAME_SHIFT NM_000378	37	Mutated in Wilm's tumor
PDGFRA	Deletion	FRAME_SHIFT NM_006206	18	PDGF signaling
PIK3R1	Deletion	FRAME_SHIFT NM_181523	16	PI3K signaling
RUNX1	Deletion	FRAME_SHIFT NM_001001890	15	Transcription factor, mutated in AML
BRCA1 (rs1799965)	SNP	nonsense	44	DNA damage response
FGFR2 (chr10:123298158:T:C)	SNP	missense	35	PI3K, FGF signaling
BRCA1 (rs1799949)	SNP	nonsense	11	DNA damage response
